# Gut microbiota changes and biological mechanism in hepatocellular carcinoma after transarterial chemoembolization treatment

**DOI:** 10.3389/fonc.2022.1002589

**Published:** 2022-10-04

**Authors:** Chao-fan Bian, Ying Wang, Ao Yu, Lulan Fu, Ding Zhang, Wenzhi Zhu, Weifu Lv

**Affiliations:** ^1^ Department of Radiology, Affiliated Provincial Hospital of Anhui Medical University, Hefei, China; ^2^ Department of Interventional Therapy, Fudan University Shanghai Cancer Center, Shanghai, China; ^3^ Department of Radiology, The First Affiliated Hospital of Anhui Medical University, Hefei, China; ^4^ Department of Medical, 3D Medicines Inc., Shanghai, China

**Keywords:** transcatheter arterial chemoembolization, intestinal permeability, gut microbiota, hepatocellular carcinoma, lipopolysaccharide, toll-like receptor 4

## Abstract

**Background and aims:**

Intestinal flora is closely associated with the occurrence and development of hepatocellular carcinoma (HCC). However, gut microbial changes and biological mechanisms in HCC after transarterial chemoembolization (TACE) treatment are rarely reported.

**Methods:**

We evaluated changes in intestinal flora after TACE in rabbit HCC models and assessed the impact of these changes on the disease. Twenty-four rabbit VX2 HCC models were established and intestinal flora structures, intestinal barrier function, changes in blood lipopolysaccharide (LPS) levels, Toll-like receptor 4 (TLR4), Cyclooxygenase-2 (COX-2), and p-signal transducer and activator of transcription 3(p-STAT3) protein expression levels were studied after TACE treatment.

**Results:**

Compared with healthy rabbits, the intestinal flora in HCC models exhibited structural changes; intestinal barrier function was decreased, and increased LPS levels entered the circulation. A short-term follow-up after TACE showed the procedure partially reversed the intestinal microflora disorder caused by the tumor: intestinal barrier and liver functions were improved, intestinal LPS levels in the blood were reduced, and liver metabolism toward LPS was enhanced. Correlation analyses of the first 75 significantly changed bacteria with clinical factors showed that harmful bacteria had decreased and beneficial bacteria increased. Blood LPS levels and downstream signaling molecule TLR4, COX-2, and p-STAT3 protein expression levels were reduced, which correlated with tumor drug resistance and invasion capabilities.

**Conclusions:**

We first characterized gut microbiota changes and biological mechanisms in HCC after TACE treatment. Our data provide a theoretical research basis for TACE combined with an intestinal flora intervention and systemic chemotherapy.

## Introduction

Hepatocellular carcinoma (HCC) is one of the most common malignant tumors in adults, accounting for one-fifth of the disease incidence in China (1). The disease incidence has also increased globally, with surgical resection considered the first line of treatment (1). However, due to its non-specific symptoms and high aggressiveness, most patients at presentation are at middle and late HCC stages, therefore tumors are not easily removed by surgery. Transarterial chemoembolization (TACE) is commonly used to treat liver cancer using localized, high concentrations of chemotherapy agents and the embolization of tumor vessels using oil iodide emulsion (1). Normal liver tissue is mainly supplied by the portal vein and easily metabolizes iodized oil. TACE is recommended by Barcelona Clinic Liver Cancer guidelines as a standard treatment for intermediate and some advanced liver cancers, while preserving normal liver tissue and controlling tumors (1).

In recent years, several studies have investigated relationships between diseases and intestinal flora. As the portal vein directs intestinal blood into the liver, this association means liver diseases are closely related to intestinal flora (1). Liver diseases are usually accompanied by varying degrees of intestinal microbiota-associated destruction, including cirrhosis, fatty liver disease, and liver cancer (1). Disordered metabolites not only promote disease occurrence, but also disease progression. After liver resection, different degrees of continuous portal vein congestion and barrier function decline increase LPS levels in the blood, and is one of the risk factors associated with liver cancer recurrence. LPS-activated signaling pathways are closely associated with increased tumor drug resistance, proliferation, and invasiveness. Therefore, regulating intestinal flora, maintaining intestinal barrier function, and reducing LPS entry into the blood are particularly important for treating liver disease (1).

Patients with liver cancer display a certain level of intestinal flora disorder. Clinically, patients undergoing TACE experience a certain degree of abdominal pain, diarrhea, and other symptoms. Few reports have outlined the impact of TACE on intestinal flora, the impact on intestinal flora over a short time, and the effects brought about by these changes.

We constructed a rabbit VX2 liver cancer model to explore changes in intestinal flora, intestinal barrier function, and serum LPS levels before and after surgery. We also investigated expression changes in TLR4, NF-κB, and STAT3 in tumor tissue and explored the impact of bacterial flora alterations.

## Materials and methods

### Establishment of the VX2 tumor model

New Zealand White rabbits (3.0–3.5 kg), provided by the Experimental Animal Center of the First Affiliated Hospital of the University of Science and Technology of China (Hefei, China), were fed and housed under standard conditions according to the National Animal Care and Use Committee. Twenty-four rabbits underwent VX2 tumor implantation in the left lobe of the liver, as previously described (2). All animals underwent imaging examinations on day 16 to measure tumor diameter. Tumors were allowed to grow until a well-demarcated solitary lesion, with a diameter of 2.0–2.5 cm was present. Then, rabbits were anesthetized *via* auricular injection of 3% sodium pentobarbital (30 mg/kg). The animal protocol was approved by the Ethics Committee of Anhui Medical University (LLSC20170125).

### TACE operation

Rabbit VX2 tumor cells were purchased from Nanjing Boshixin Company. VX2 liver cancer was induced in rabbits by a modified open puncture inoculation. All 24 rabbits were divided into four experimental groups; 1) Healthy control (HC), 2) 1-day TACE (TACE on day 1), 3) 5-day TACE (TACE on day 5), and 4) 10-day TACE (TACE on day 10) (n=6 in each group). TACE was performed by infusing a 0.6 mg/kg Pirarubicin (THP) suspension (Shenzhen Main Luck Pharmaceuticals, Shenzhen, China) and 0.2 mL iodized oil (Yantai, Luyin, Shandong, China) into the left hepatic artery until the entire dose was delivered or near stasis was achieved. The HC group received sterile physiological saline as a placebo. Serum and fecal samples were obtained before surgery and at corresponding time points after surgery. Thereafter, all animals were humanely sacrificed at the corresponding time point (TACE on day 1, TACE on day 5, and TACE on day 10) using 3% sodium pentobarbital. Tumor, liver tissue, and fecal samples were immediately removed, rinsed in ice-cold normal saline, and stored at -80°C until further analyses.

### Enzyme linked immunosorbent assay (ELISA)

Rabbit diamine oxidase (DAO), alanine aminotransferase (ALT), D-lactic acid (D-LA), Toll-Like Receptor 4 (TLR4), and lipopolysaccharide (LPS) levels in serum were detected, and analyzed according to manufacturer’s instructions, using a rabbit DAO ELISA kit (JYM0207Rb, Jiyinmei, Wuhan, China), rabbit D-LA ELISA kit (JYM0092Rb, Jiyinmei), ALT ELISA kit (C009-2-1, Jiancheng, Nanjing, China), rabbit TLR4 ELISA kit (JYM0208Rb, Jiyinmei), and end-point chromogenic Tachypleus Amebocyte Lysate2 kit (Xiamen Bioendo Technology, EC10545S, Fujian, China), respectively.

### Western blotting

VX2 tumor cells from rabbits were lysed in RIPA buffer (Beyotime, Shanghai, China). Total protein samples (30 μg) were separated using sodium dodecyl sulfate-polyacrylamide electrophoresis (Solarbio, Beijing, China) at a separation voltage of 120V for 1 h and transferred to polyvinylidene difluoride membranes (Solar). Membranes were blocked in 4% bovine serum albumin, washed, and incubated with a primary antibody at 4°C overnight. Antibodies were: mouse anti-β-actin (sc-47778, Santa Cruz Biotechnology); rabbit anti-NF-κB p65 (#4764, Cell Signaling Technology (CST)); mouse anti-STAT3 (#9139, CST); rabbit anti-p-Tyr705-STAT3 (#9145, CST); rabbit anti-TLR4 (PL0402123); and rabbit anti-COX-2 (ab102005, Epitomics). Membranes were washed three times in phosphate-buffered saline (PBS) for 10 min each time, incubated with secondary antibodies for 2 h, and washed as described. Finally, protein bands were detected using a chemiluminescent blotting analysis system (Thermo Fisher Scientific, Waltham, MA, USA).

### Total RNA extraction, RT, and quantitative-PCR (q-PCR)

Residual tumor tissue at tumor margins was excised and stored in liquid nitrogen or -80°C. RNA was extracted from tissue as follows: (1) 50–100 mg of tissue was cut into pieces, ground in liquid nitrogen, and 1 mL TRIzol (Life Technologies, 204403, Shanghai, China) was added to the homogenate. (2) Samples were centrifuged at 12000 rpm for 10 min at 4°C to remove cellular debris. (3) Next, 0.2 mL chloroform was added, tubes shaken vigorously for 15 s, and then incubated at room temperature for 3 min. (4) Tubes were centrifuged at 12000 rpm at 4°C for 15 min, and an appropriate volume of supernatant (approximately 500 μl) was added to another tube. (5) Next, 0.5 mL isopropyl alcohol was added and mixed well under mild conditions. (6) Tubes were then incubated at room temperature for 10 min, centrifuged at 12000 rpm at 4°C for 10 min, and supernatants discarded; (7) Then, 1 mL 75% ethanol (in DEPC water) was added, tubes centrifuged at 12000 rpm at 4°C for 5 min, and supernatants discarded. (8) The RNA precipitate was dried at room temperature for 30 min. (9) Then, 20–50 μL DEPC water was added, the RNA precipitate dissolved at 55°C for 10 min, and tubes were stored at -80°C.

RT reaction steps: (1) Total RNA (1 μg), 10 μM Oligo (dT), and 1 μL DEPC water to 12 μL were added to a 0.2 ml tube, mixed gently, and pulse centrifuged. (2) The PCR instrument was heated to 65°C for 5 minutes and then iced and bathed for 3 minutes immediately. (3) 5× Reaction Buffer 4.0 μL, 10 mM dNTP Mix (2 μL), RibolockTM Rnase inhibitor (1 μL), and RevertAidTM M-Mulv Reverse were added into the EP tubes Transcniptase 1 mu L. (4) 42°C, 60 min, 70°C, 5 min. (5) The cDNA was removed and stored at -80°C.

Total RNA was extracted from tumor tissue, cells, or paired adjacent tissue using TRIzol. A PrimeScript™ RT reagent kit (Thermo Scientific, K1622) was used to synthesize complementary DNA (cDNA), and qPCR was conducted using the TB Green™ Fast qPCR mix kit (Takara, Japan). Glyceraldehyde 3-phosphate dehydrogenase was used as an internal reference. Primers are listed (**Table 1**).

**Table 1:** Primers.

Gene	Amplicon Size (bp)	Forward primer (5'→3')	Reverse primer (5'→3')
GAPDH	98	CACCCACTCCTCTACCTTCG	TGCTGTAGCCAAATTCGTTG
TLR4	151	TGCTTCTAGTTGGCCGAAGA	TAGTGAAGGCAGAGCCGAAA
COX-2	149	GATGATCTACCCGCCTCACA	CTGGATGCTCCTGTTTGAGC

### Immunohistochemistry

Sections were dewaxed, dehydrated, rehydrated before immunostaining, and sealed with goat serum. Then, antibodies against COX-2 (#12282, CST), GAPDH (sc-25778, Santa Cruz Biotechnology), or TLR4 (sc-10741, Santa Cruz Bio) were added. Slices were dewaxed in xylene and dehydrated in ethanol. After endogenous peroxidase neutralization and the microwave extraction of antigens, slices were blocked in goat serum, then incubated overnight in primary antibodies against COX-2 (#12282, Cell Signaling Technology), GAPDH (sc-25778, Santa Cruz Biotechnology) or TLR4 (sc-10741, Santa Cruz Biotechnology). Sections were washed in PBS and incubated with a universal secondary antibody (PV-6000, Zsbio, Beijing, China) for 30 min, after which 3-3 diaminobenzidine (DAB) and hematoxylin were applied. After washing in PBS containing 0.05% Tween 20, sections were incubated with a general purpose second antibody (PV-6000, Zsbio, Beijing, China) for 30 min. Reaction products were observed using 3-3 diaminobenzidine (DAB) and stained with hematoxylin. Dyed area calculations were performed in Image-pro Plus 6.0 software and the integrated optical density (IOD) analysis of three regression fields was performed. Protein expression was expressed as average density, which was total IOD/area.

### Tunel staining liver samples

The DeadEnd fluorescent TUNEL system (Promega, Madison, WI, USA) was used for TUNEL analyses of paraffin-embedded fixed tumor sections. Dewaxed sections were equilibrated in PBS, permeated by protease K, fixed in 4% paraformaldehyde, and mixed in the dark at 37°C with a TdT reaction for 1 h. Sections were washed in 2×SSC buffer (0.3 M NaCl and 30 mM trisodium citrate, pH 7.0), counterstained with propidium iodide (PI) (1 μg/mL), and covered with Vectashield fixation medium. Images were captured with a fluorescence microscope (Motic BA410E, Xiamen, China) and showed red apoptotic cells and blue nuclei.

### Hematoxylin and eosin (H&E) staining of liver samples

H&E staining was performed to visualize the tumor and normal tissue structures. Briefly, all tissues after resection were fixed in 15% formalin, embedded in paraffin wax, sectioned into 4 µm slices, stained with H&E, and visualized (Olympus, Japan).

### Microbial bioinformatics analysis

Feces from experimental rabbits were collected under aseptic conditions and stored at -80°C. After thawing, 0.2 g of feces were processed using TIANamp bacterial DNA extraction kits to extract total feces DNA for gene sequencing and bioinformatics. Briefly, the sequencing process comprised the following steps: (1) One end of the DNA fragment was complementary to the primer base. Target fragment fixations; (2) PCR amplification produced DNA clusters. (3) DNA amplicon was linearized to single strands; (54 fluorescent-labeled dNTP and modified DNA polymerase were added. This process synthesizes only one base per cycle. (5) The nucleotide class of the first round of reaction polymerization of the template sequence was read according to the reaction system. (6) Chemical cutting of “fluorescence” and “termination groups” was conducted to restore the viscosity of the 3 ‘end and continue to polymerize the second nucleotide. (7) A template DNA fragment was obtained based on fluorescence signal results.

### Biological information processes

PE reads from Miseq contained barcodes to distinguish samples. After quality control and filtering for sequence quality, splicing according to overlap relationship, and another round of quality control and filtering splicing sequences, optimized sequences were finally generated. These sequences were analyzed by systematic level and cluster analysis to generate OTU (Operational Taxonomic Units). According to OTU cluster analysis results, multiple diversity index analysis and sequencing depth detection were performed on OTU.

Community structures and taxonomic information were statistically analyzed at each systematic level, and β-diversity(Bray-curtis) was analyzed between samples. Based on these steps, community structures and phylogeny were examined.

### Taxonomy profiling, community diversity estimations, and *de novo* assembly

An equal number of sequence reads were used to analyze microbial diversity and richness to minimize the impact of random sequencing errors. The Mother V. 23.0 program was used to cluster sequences as OTUs at a 3% distance. Sequences were systematically classified using the SILVA rRNA database (https://www.arb-silva.de/). All data were analyzed by Microgene Biotechnology LTD.

### Statistical analyses

Statistical analysis was performed using SPSS 20.0 (SPSS Inc.). Graphs were made using Graph Pad Prism version 6.00 (Graph Pad Software). Analysis of variance (ANOVA) was performed using SPSS 20.0. The mean density of each section was analyzed in Image J software. All values were presented as the mean ± standard deviation for three or more independent experiments. The Student t-test was used to examine differences between the two groups. A p<0.05 value was considered statistically significant. Correlations between OTU abundance were evaluated by Spearman correlation analysis in Graph Pad Prism 6.

## Results

### Study overview

**Figure 1:**
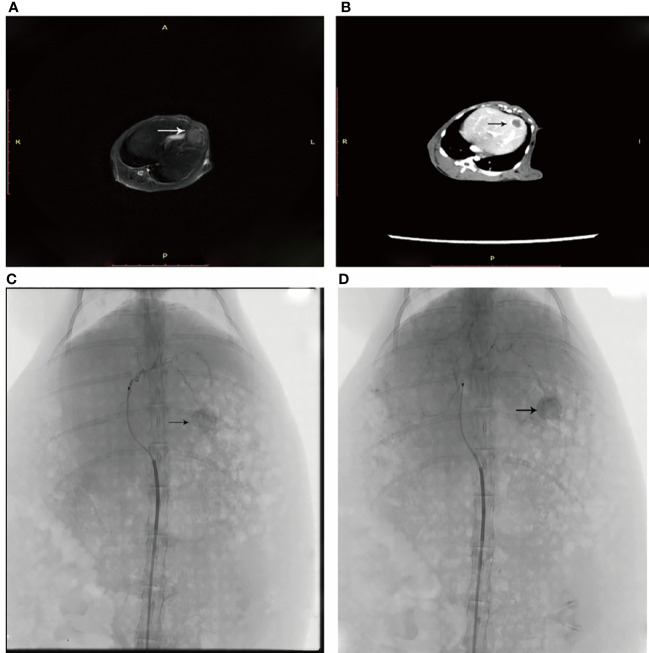
**(A)** An arrow shows the left lobe of the liver where the neoplasm is in T2 sequence. **(B)** Enhanced CT shows the tumor in the left liver leaf. **(C)** DSA angiography shows abundant blood supply to the tumor. **(D)** An iodized oil deposit is shown by the arrow.

Eighteen (18) study rabbits underwent VX2 tumor implantation in the left lobe of the liver. Tumor growth rates were observed and macroscopic images indicated isolated tumors on day 16 (**Figure 1**). Rabbits were then randomly assigned to four groups of six animals (groups 1 d, 5 d, 10 d, and HCs). For histological examinations, rabbits were sacrificed at the corresponding time point.

### Sequencing characteristics

In total, 42 fecal samples including control group, experimental group preoperative and postoperative samples were collected and DNA underwent high-throughput 16S rRNA amplicon sequencing. After assembly, quality control, and filtering singletons, 1,458,759 high quality sequences were obtained. Each fecal sample eventually yielded > 30,000 high quality sequences. Sequences with similarities ≥ 97% were classified and analyzed, and OTUs were classified into different species levels for analysis. Thus, the 1318 OTUs generated by random reading covered almost all sequences. The abundance ratio of bacteria is shown (**Figures S1A, B**).

### The classification and composition of intestinal flora in tumor models

**Figure 2:**
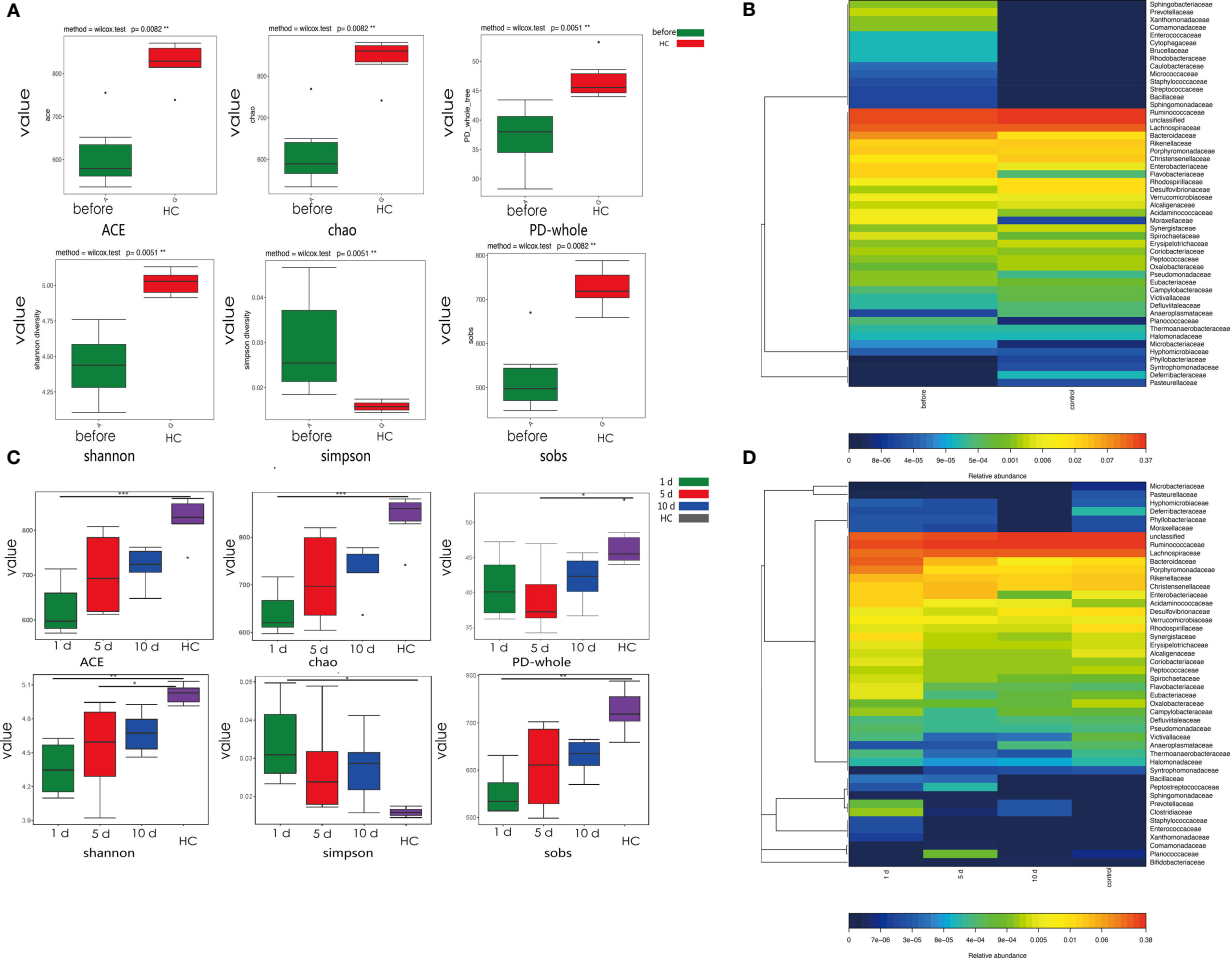
Phylogenetic diversity of gut microbiota in tumor models compared with HCs at different time points. **(A)** Alpha diversity in tumor models and HCs. Box plots depict greater diversity in HCs compared with tumor models, according to ACE, Chao, and Shannon indices. Horizontal lines in box plots show median values; upper and lower ranges in boxes show 75% and 25% quartiles. **(B)** Heat map comparisons in tumor models and HCs. **(C)** One, 5, and 10 days post-surgery, α-diversity in tumor models and HCs. Box plots depict greater diversity in HCs when compared with tumor models, according to ACE, Chao, and Shannon indices. Horizontal lines show median values; upper and lower ranges in boxes represent 75% and 25% quartiles. **(D)** Heat map comparisons at different time points in models and HCs. ***p < 0.001; **p <0.01; *p < 0.05.

We observed changes in gut microbial diversity in tumor models when compared with HCC models. Different diversity indices (ACE, Chao, PD whole tree, Shannon diversity, Simpson diversity and SOB) were used to evaluate intestinal microbial diversity. Gut microbial diversity, using ACE, Chao, PD whole tree, Shannon diversity and SOB (**Figures 2A–D**) was lower in tumor models when compared with HCs (*p*=0.015, 0.015, and 0.0069, 0.00097, 0.0085, respectively), while opposite changes were observed in the Simpson’s diversity index (*p*=0.0037).

### Changes in intestinal flora composition in tumor models

**Figure 3:**
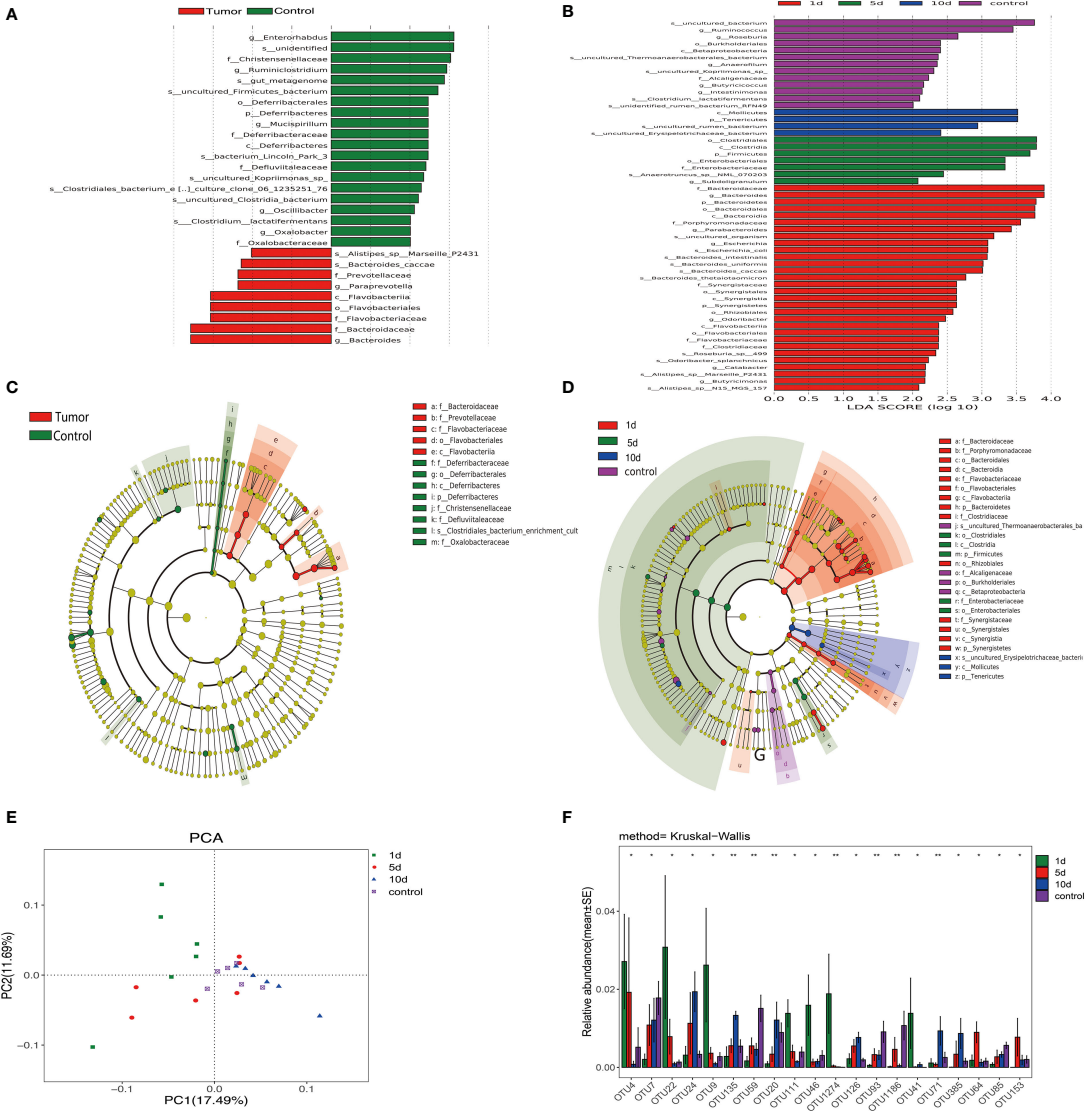
Linear discrimination analysis effect size (LEfSe) and linear discriminant analysis (LDA) revealed differences in taxonomic composition in tumor models when compared with HCs, and changes in intestinal microflora composition in tumor models before and after transcatheter arterial chemoembolization (TACE). **(A)** LDA scores showing differences in models (red) and HCs (green). **(B)** A cladogram showing taxonomic compositions in models (red) and HCs (green). **(C)** Bacterial levels in models underwent TACE at different time points and HCs in LDA scores. **(D)** A cladogram showing different taxonomic compositions in models at different time points and HCs. One day after TACE (red), 5 days after TACE (green), 10 days after TACE (blue), and HCs (purple). **(E)** Principal component analysis (PCA) of intestinal flora in animals undergoing TACE at indicated time points and HCs. **(F)** Bacterial diversity analyses in groups undergoing treatment. **p <0.01; *p < 0.05.

We assigned 98.3% and 91.6% of all reads into class and order categories, respectively (**Figure 3A** and supporting information). *Firmicutes, Bacteroidetes, Tenericutes*, and *Proteobacteria* were the most abundant entities (**Figure 3B**). In addition, *Ruminococcaceae, Lachnospiraceae, Bacteroidaceae*, and *Rikenellaceae* dominated intestinal flora at the family level (**Figure 3C** and **Figures S1A, B**). We observed a higher abundance of Bacteroidetes in tumor models, while Firmicutes were enriched in HCs. *Bacteroidaceae, Prevotellaceae, Flavobacteriaceae, Flavobacteriales*, and *Alistipes_sp_Marseille* abundance rates were higher in tumor models than HCs, while *Ruminiclostridium, Christensenellaceae, Enterorhabdus, Christensenellaceae*, and *Mucispirillumgenera* were enriched in HCs (**Figures 3A, B**).

Furthermore, we validated results from LEfSe analyses by permutational ANOVA analyses and observed that *Bacteroidaceae*, and *Flavobacteriaceae*, *Prevotellaceae*, and *Flavobacteriia* genera were highly substantiated in tumor models, while the *Clostridiales_bacterium* phylum, and *Oxalobacteraceae*, *Defluviitaleaceae*, and C*hristensenellaceae* genera were coincidentally higher in HCs. Principal component analysis (PCA) of microbiota also showed that the microbiota of different group clearly differed (**Figure 3E**).

### Dynamic changes in intestinal flora in different subgroups undergoing TACE

**Figure 4:**
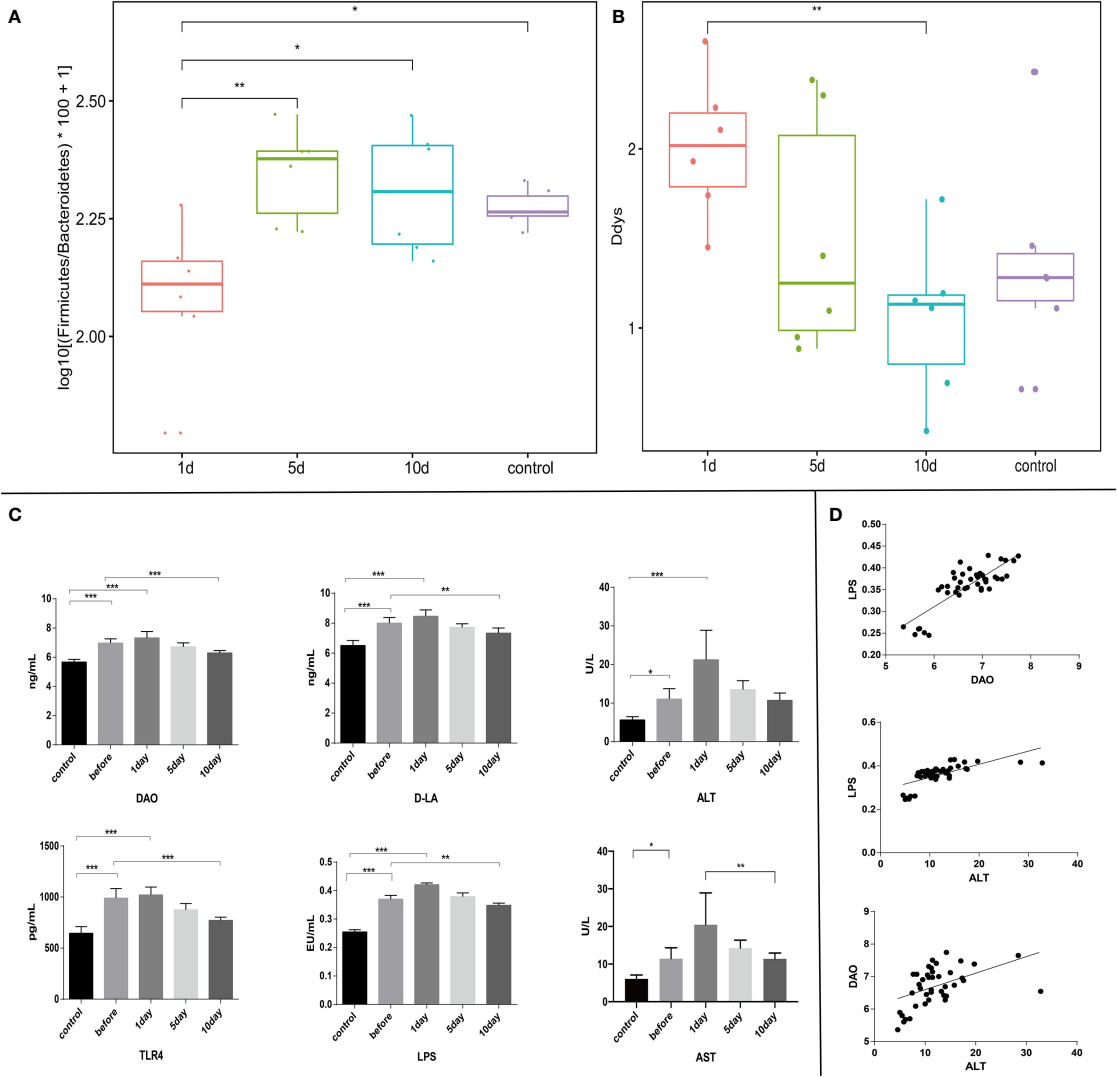
**(A)** and **(B)** Different evaluation methods were used to measure disturbance levels in intestinal flora (3). Intestinal flora disturbance peaked on the day after surgery, with no significant differences in intra-group comparisons. **(C)** Serum LPS, TLR4, DAO, D-LA, ALT, and AST levels. **(D)** Correlation analyses between serum levels of DAO and LPS (*r*=0.82, *p ≤* 0.05), ALT and LPS (*r*=0.68, *p ≤* 0.05), and ALT and DAO (*r*=0.48, *p ≤* 0.05). ***p < 0.001; **p <0.01; *p < 0.05.

Fresh feces from 18 tumor models from the three tumor groups on days 1, 5, and 10, and six animals from HCs were gathered. Different diversity indices [Abundance-based Coverage Estimator (ACE), Chao, PD whole tree, Shannon diversity, Simpson diversity, and Shannon, and observed richness (Sobs)] were used to assess gut microbiota diversity. At different treatment time points, different intestinal flora bacterial species showed dynamic changes. As shown (**Figures 4A, B**), the dysbiosis index peaked on the group 1 day underwent TACE. Furthermore, validation results from LDA effect size (LEfSe) analysis and linear discriminant analysis by the permutational ANOVA test (P < 0.05, linear discriminant analysis [LDA] score > 2; **Figures 3A, B**). *Enterorhabdus* HCs, *Chistensenellaceae*, and *Ruminiclostridium* displayed higher abundance, tumor model *Alistipes_sp_Marseille_p2431*, *Bacteroides_caccae* and *Prevotellaceae* showed high abundance. Postoperative bacteria abundance presents the dynamic change, 10 dmollicutes postoperatively, *Tenericutes* abundance show a significant increase. These results showed a variety of intestinal flora undergoing dynamic changes. Variable bacterial levels at different time points were further evaluated for significant differences using differentially abundant features, multiple hypothesis testing, and false discovery rate analysis of rare frequency data. We observed that *Firmicutes*, *Bacteroidetes*, *Tenericutes*, *Synergistetes* at the phylum, and *Clostridiales*, *Bacteroidales*, and *Enterobacteriales* at the order, and many other bacterial genera were manifested as changes beginning one day after TACE and tending to change with time in the sample of the HCs (**Figures 3C, D, F**).

### Functional changes associated with TACE

**Figure 5:**
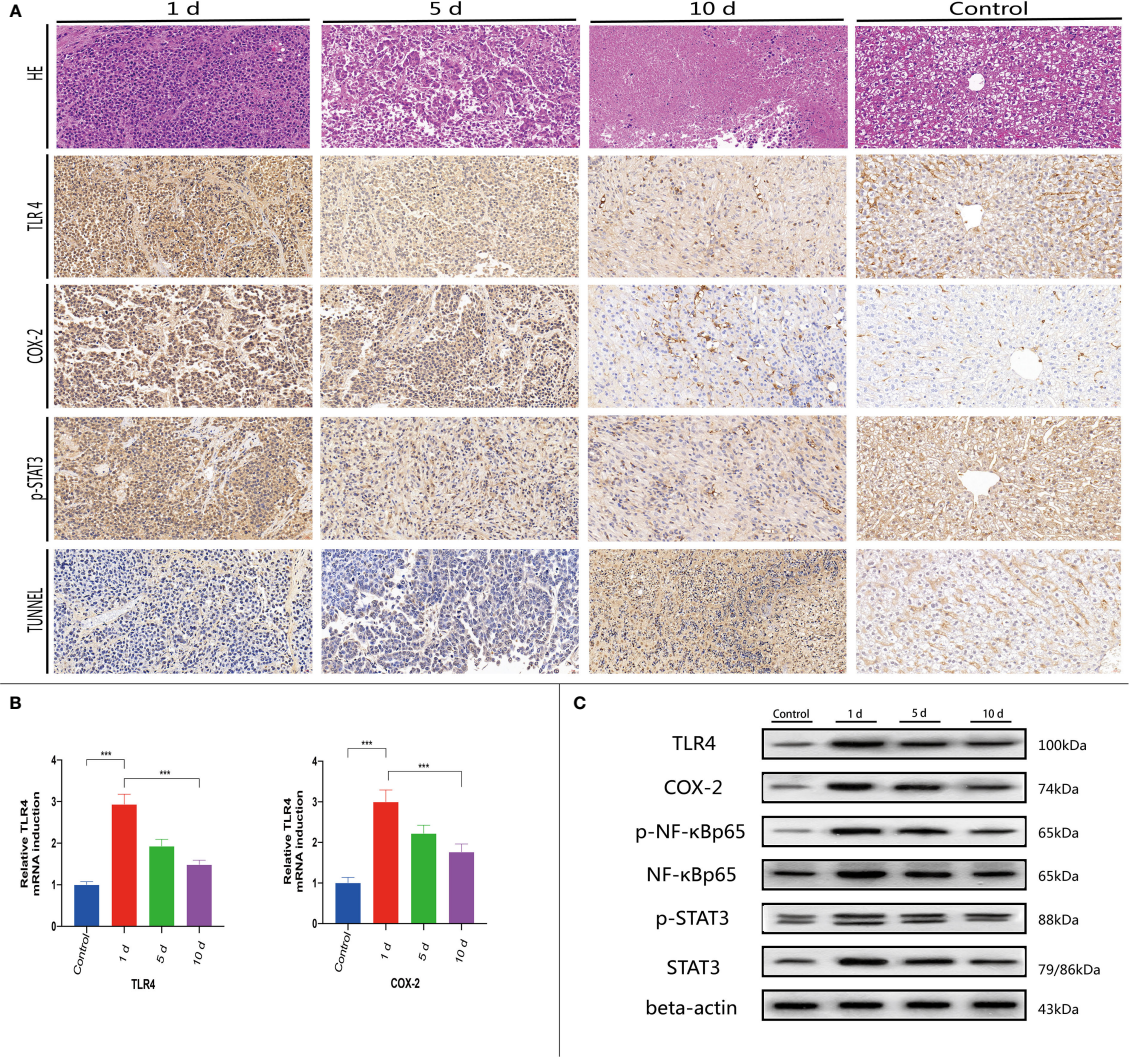
At 1, 5, and 10 days post-TACE, at each time point, animals were sacrificed with a pentobarbital overdose. **(A)** Histological H&E staining in tumor tissue (magnification ×200); IHC staining of TLR4, p-STAT3, COX-2, and TUNEL in tumors (magnification ×200). **(B)** Quantitative PCR (qPCR) was used to detect TLR4 and COX-2 mRNA levels in tissue. Data are represented as the mean ± standard deviation (SD). ^∗∗∗^ *p* < 0.001. **(C)** Western blotting of tumor tissues.

Experimental rabbits were injected with an overdose of barbiturate at corresponding time points after TACE. Blood samples and tumor tissue were collected from tumor margins after euthanasia. Serum LPS, TLR4, DAO, D-LA, ALT, and AST (aspartate aminotransferase) levels were investigated. Tumor sections were stained using the TUNEL assay and H&E staining. The plasma concentrations of these acute phase reactant showed a typical abrupt increase after TACE (**Figure 4C**), peaking at day 1 and decreasing with time (**Figures 4C**, **Figures 5B, C**). H&E staining and the TUNEL assay were performed to identify pathological changes and apoptosis status in sections (**Figure 5A**).

### TLR4, p-STAT3, and COX-2 levels are downregulated in normal flora

Previous studies indicated that many immune cells, such as dendritic cells and macrophages, effectively express TLRs. In addition to their presence on the surface of normal cells, TLRs are also expressed on tumor cells and appear to regulate tumor immunity and participate in tumor biological functions. Recent research on the hepatointestinal axis indicated that intestinal endotoxin pathogen-related molecular patterns (PAMPs) entered the liver *via* the portal system, with endotoxins activating TLR4 expression and up-regulating TLR4 receptors in tissues, mainly *via* the MYD88-dependent NF-κB pathway. Similarly, endotoxin activation of TLR4 expression may also lead to interferon regulatory factor (IRF) pathway activation which is not MYD88-dependent. NF-κB pathway mechanisms have been widely studied. Serum LPS was significantly increased in early stages post-TACE (**Figure 4C**), therefore, immunohistochemical staining (**Figure 5A**) was used to investigate *in situ* COX-2, TLR4, and p-STAT3 expression in tumors and western-blot showed that the expression of the detected protein was lower in the 10d group than in the 1d group.(**Figure 5C** and **Figure S2**)

### Gut microbiota structure are partially reversed at 10 days in animals receiving TACE

**Figure 6:**
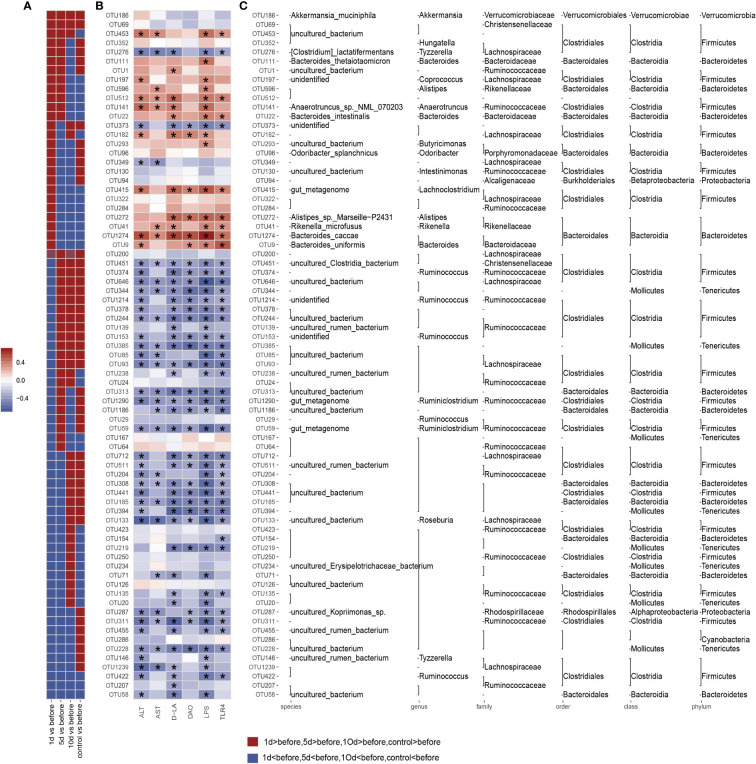
Correlation analysis based on flora operational taxonomic units (OTUs) and clinical factors. The color intensity indicates the correlation degree between OTU abundance and principal parameters which were evaluated by Spearman’s correlation analyses. The top 75 OTUs, with statistically significant abundance differences by Spearman’s correlation analyses with specific metabolic parameters in groups. Significantly different clinical factor changes are marked with an asterisk (*) (p < 0.05). **(A)** OTUs abundance changes. Red and blue showing the OTUs abundance with more and less abundant in HCs and postoperative group in different time window groups relative to the before group. **(B)** Correlations between 75 OTUs and host metabolic parameters. Color intensity represents the level of association between OTU abundance and principal parameters, as assessed by Spearman's correlation analyses. Rows correspond to OTUs with identities on the left, and columns correspond to serum parameters. Positive and negative associations are shown in red and blue. The asterisk (*) indicates significant difference (p < 0.05). **(C)** OTU taxonomy is shown on the right. OTUs whose abundance was altered by tumor models and then significantly reversed by transcatheter arterial chemoembolization (TACE).

We also assessed gut microbiota associations with clinical parameters. Bacterial phylogenetic types were identified (**Figures 4B-D**) by ANOVA before and after TACE, and also in HCs. Of those species with significantly different abundance changes (*p*<0.05), the top 75 were screened. To assess the potential relationship between alterations in gut microbiota composition, including liver function, intestinal permeability, lipopolysaccharide, and TLR4, we used Spearman’s correlation analysis. 15 key OTUs were positively correlated with at least one physiological index (**Figure 4D**), including *Bacteroides*, *Rikenella*, *Alistipes*, *Anaerotruncus*, etc. Also, 42 key OTUs were negatively correlated with at least one physiological index, including *Mollicutes*, *Ruminococcaceae* and *Lachnospiraceae*, etc. Notably, 45 key OTUs were higher and 30 key OTUs lower in HCs when compared with tumor models before surgery. Also, 33 key OTUs were negatively correlated with at least one clinical parameter in 45 key OTUs (**Figure 6**), while 24 key OTUs were significantly positively correlated in these 33 key OTUs. Up until 10 days post-surgery, key OTUs decreased from 15 to 2. Also, 26 key OTUs were negatively correlated with clinical factors and were higher when compared with fecal samples before surgery (**Figure 6**).

## Discussion

We characterized the gut microbiota in tumor models in comparison with HCs, before and after TACE treatment. We also investigated associations between intestinal flora, clinical parameters, and dynamic changes during treatment. Our results demonstrated that classified composition and gut microbiota diversity were significantly different in tumor models. The intestinal flora composition of models and HCs was dominated by *Bacteroidetes* and *Firmicutes*. Also, we determined that intestinal flora exhibited specific change patterns at different time points after treatment.

We observed a higher gut microbiota diversity in HCs when compared with tumor models, however, TACE did not change diversity within treatment groups. *Firmicutes*, *Bacteroidetes*, *Tenericutes*, *Synergistetes*, and other bacterial levels indicated that as time progressed post-TACE, bacterial abundance was closer to pre-TACE HC levels. *Ruminococcus*, *Roseburia*, and other strain levels showed an upward trend at 10 days post-surgery when compared with day 1 after surgery, and the HC group was higher than the tumor group. *Bacteroides*, *Parabacteroides*, *Escherichia* and other strains showed a downward trend at 10 days after surgery compared with 1 day after surgery, and the HC group was lower than the tumor group. Differential analyses based on OTUs indicated that microflora showed a trend towards the abundance level of the HC group with the prolongation of TACE time. *Ruminococcus* is a widely studied strain whose main role is to convert primary bile acids into secondary bile acids. In patients with colorectal cancer and ulcerative colitis, the abundance of flora was significantly decreased (4). *Bacteroides* participate in many important metabolic activities in human colon, including carbohydrate fermentation and utilization of nitrogen-containing substances, and play an important role in maintaining the homeostasis of intestinal flora (5). There is a certain degree of increase in abundance under pathological conditions, including inflammatory bowel disease and liver cancer, which are also gram-negative bacilli and are related to the production of toxoid. This study suggests that the abundance of *Ruminococcus* in the tumor model is significantly lower than that in the normal control, and bacteroides in the tumor model is higher than that in the control group. Several other gut microbiota also show significant changes in rabbits underwent TACE. The follow-up after TACE suggests that this abundance control can be partially reversed, and a variety of bacteria show similar changes, which further proves that the intervention of intrahepatic diseases can change the changes of intestinal microbial structure.

To explore the effects of intestinal flora changes and possible liver-gut axis mechanisms during TACE (6, 7), we studied serological indicators in rabbits. When compared with the HCs, preoperative serum ALT levels in models were higher, possibly from invasion and damage due to tumor tissue in the liver (8). In terms of intestinal permeability, DAO is released into the blood when intestinal mucosa becomes ischemic, congested, and damaged (9–11). D-LA is mainly produced by intestinal microorganisms, which are produced by the metabolic lysis of intestinal microorganisms (12). When intestinal flora overgrowth and mucosal barrier functions are decreased, blood inflow rapidly increases. Therefore, DAO and D-LA are ideal indicators that reflect intestinal mucosal mechanical barrier integrity and the degree of damage (13). Mechanical barrier integrity is key to intestinal mucosal barrier function and a most critical link (14). We noted that preoperative DAO and D-LA levels in model animals were higher than HCs, suggesting liver lesions had affected intestinal permeability. TACE is the preferred treatment for unresectable liver cancer, however the procedure inevitably causes ischemia and hypoxia in the tumor and surrounding liver tissue (15), leading to hepatocyte damage and transient increases in blood ALT levels. Chemotherapeutics can also increase apoptosis in normal tissue, but this liver damage normally recovers within 3–10 days (16). In the hepatointestinal circulation, whether the liver cancer can be effectively controlled, whether the liver function can be recovered and improved after treatment, thereby improving the status of the intestine?

In model rabbits after TACE, we found that DAO, ALT and D-LA levels were higher in the 1 day TACE group than before treatment, but with the effective control of tumors, compensation and recovery of liver function, improvement of intestinal barrier function, ALT began to decline in the group 5-day after TACE, lower than that in the group 1 day after TACE, and DAO and D-LA began to decline. DAO and D-LA levels in the 10-day group after TACE were lower than levels before TACE, and slightly higher than the HC group. Correlation analyses of ALT and DAO, and ALT and D-LA showed good correlations and reflected the close links between hepatointestinal function and HCC.

Our correlation analyses of clinical factors with intestinal flora indicated that bacterial species numbers were positively correlated with ALT, DAO, D-LA, LPS, and TLR4 increases, and that numbers of bacterial species negatively correlated decreased in the comparison of different time windows before and after surgery. *Bacterial* species negatively correlated with clinical factors in the HC group were higher than those in the tumor group and the 10-day group after TACE. These observations suggested TACE partially reversed intestinal flora disorders caused by tumor invasion, and improved intestinal flora status.

Increased attention has focused on the role of TLR4 and LPS in tumor development, and endotoxin as an important mediator of the interaction between the liver and intestinal axis (17, 18). Liver disease causes intestinal flora disorders which in turn induce intestinal endotoxemia, thereby forming a vicious circle. Recent liver-gut axis studies reported that intestinal endotoxins, such as PAMPs (19), enter the liver *via* the portal vein system to upregulate TLR4 activation (20). Lin et al. showed that the LPS-ligand of TLR4 activated the STAT3 pathway which activated the COX-2/PGE2 signaling axis, leading to hepatoma cell proliferation, and increasing drug resistance and tumor invasiveness (21). Dapito et al. reported that endotoxins and TLR4 were important pathogenic factors in the liver-gut axis in pathological conditions, thereby affecting tumor prognosis (22). Previous studies indicated that tumor cells inhibited the body’s immune defenses by expressing TLR4 and activating downstream signaling molecules to construct a local tumor microenvironment (23).

Our LPS and TLR4 investigations showed that change trends in LPS in TLR4 expression were consistent with change trends in liver function and TLR4 function. Serum LPS in the day 1 group post-surgery were higher than preoperative levels. Serum LPS in the day 5 group post-surgery were decreased, whereas serum LPS in the day 10 group post-surgery were significantly lower than preoperative levels (*p*<0.05). Our analyses also indicated that DAO and LPS, and DAO and TLR4 displayed good correlation levels, which suggested that increased LPS in the blood could promote TLR4 expression *in vivo*, with increased intestinal permeability. Correlation analyses of ALT and LPS showed good correlations between liver function and LPS; when ALT was low, LPS was low. Studies have shown that liver disease can cause metabolic disorders in the liver and block intestinal blood reflux, resulting in the decline of intestinal mucosal barrier function. We observed damage and invasion of tumor tissue to normal liver tissue, as well as the invasion and compression of liver blood vessels, portal venous return is blocked, resulting in intestinal mucosal congestion and edema, affecting intestinal mucosal barrier function, increased endotoxin transfer, improved blood return and restored intestinal mucosal barrier function after effective tumor control. LPS induces nitric oxide and hydrogen peroxide production, activates redox-sensitive transcription factors such as tumor necrosis factor-α (TNF-α), alters intestinal mucosa structures, and increases intestinal permeability. When intestinal permeability increases, bacterial and LPS migration also increases. Endotoxemia exacerbates LPS-mediated inflammation, further damaging liver tissue, and the two reinforce each other. After TACE, LPS levels were reduced by tumor control, recovery of liver function increased clearance of intestinal toxins such as LPS, improved intestinal mucosal barrier function, decreased bacterial transmigration and entry into blood, and recovery of liver function. This meant that conventional TACE improved liver and gut function, reduced LPS displacement, helped recover liver function, increased LPS degradation, coordinated the *in vivo* regulation of tumor signaling pathways, play a similar depletion TLR4 effect, reduced the release of cytokines and inflammatory medium caused by chronic inflammation of liver cell damage, and then together play the role of tumor treatment.

NF-κB upregulates the expression of many inflammatory cytokines, including TNF-α, interleukin-6 (IL-6), and IL-8, and is an important downstream inflammatory signal of TLR4 (20, 24, 25). Studies indicated that STAT3 was highly expressed in several tumor tissues and cell types (26), and also showed the same characteristics in liver cancer cells. COX-2 by regulating the synthesis of prostaglandins in the body to regulate inflammatory reaction. COX-2/PGE2 signal axis abnormal activation is also regarded as the important symbol of the tumor, can cause local inflammation, to participate in the construction of tumor microenvironment (27). Prostaglandin E2 (PGE2) activates STAT3 by binding to its receptor and mediating downstream signal cascade transduction. Previous studies reported that both LPS and TLR4 showed a downward expression trend 10 days after TACE (1). We detected the above key signaling proteins and also showed the same change pattern as LPS, suggesting that TACE can affect tumor cells through this signaling pathway after regulating intestinal function by affecting intestinal flora and depleting LPS and TLR4.

## Limitations

Our sample size was small, therefore, numbers must be increased in future studies to improve data/result reliability. In addition, our liver cancer model was not complicated with viral hepatitis and cirrhosis, and did not completely simulate the human disease state. VX2 hepatocellular carcinoma model can better simulate the effect of human interventional embolization surgery, which is the first choice of animal model for interventional radiology study. However, it is difficult to fully match the characteristics of human liver cancer. The current liver cirrhosis model uses carbon tetrachloride as the induction reagent, while it will inevitably have a serious impact on the intestinal flora and the overall condition of animals, and affect the study of the relationship between tumor and intestinal flora. This study mainly studied the dynamic changes of intestinal microbiota after embolization, and did not focus on the effects of liver cirrhosis and hepatitis on the body. Cirrhosis and HCC models must be established in the future. Also, fecal sample flora analysis can reflect intestinal flora status to some degree, but it was difficult to fully represent this intestinal flora in our model.

## Conclusions

Our data indicated the gut microbiota in liver tumor models were significantly changed, and could be related to multiple factors, including liver metabolism, intestinal venous reflux, and intestinal barrier function. TACE partially reversed intestinal microflora structures, improving intestinal barrier functions, and reducing LPS levels in blood. Investigations on signal molecules downstream of LPS suggested TACE indirectly affected tumor therapy *via* the liver-gut axis. Furthermore, we provided new insights for HCC clinical treatment, including combined chemotherapeutics with TLR4 inhibitors, intestinal flora interventions, and/or intestinal mucosal protection. These strategies may enhance TACE efficacy and improve prognoses.

## Data availability statement

The datasets presented in this study can be found in online repositories. The names of the repository/repositories and accession number(s) can be found below: SRP346000, PRJNA780246, NCBI.

## Ethics statement

The animal study was reviewed and approved by LLSC20170125.

## Author contributions

Study conception and design, WL and CB. Conducted experiments, WL, CB, and YW. Contributed to data acquisition, WL and YW. Analyzed data, WL, YW, and WZ. Manuscript editing, CB, LF, and DZ. All authors contributed to the article and approved the submitted version.

## Funding

This study was funded in part by supported the Fundamental Research Funds for the Central Universities (WK9110000061).

## Conflict of interest

DZ was employed by the company 3D Medicines Inc.

The remaining authors declare that the research was conducted in the absence of any commercial or financial relationships that could be construed as a potential conflict of interest.

## Publisher’s note

All claims expressed in this article are solely those of the authors and do not necessarily represent those of their affiliated organizations, or those of the publisher, the editors and the reviewers. Any product that may be evaluated in this article, or claim that may be made by its manufacturer, is not guaranteed or endorsed by the publisher.

## Supplementary material

Supplementary Figure 1
**(A, B)** Clustering tree Based on the Weighted UniFrac distance in phylum and order level. **(C)** Analysis of different species of microbial.

Supplementary Figure 2 Relative protein expression. Data are represented as the mean ± standard deviation (SD). ***p <0.001; * *p <0.01; *p <0.05.
