# Supplementary figures and images for: Gut microbiota changes and biological mechanism in hepatocellular carcinoma after transarterial chemoembolization treatment
Source: Front Oncol. 2022 Oct 4;12:1002589. doi: 10.3389/fonc.2022.1002589 (PMC9577458; doi:10.3389/fonc.2022.1002589)

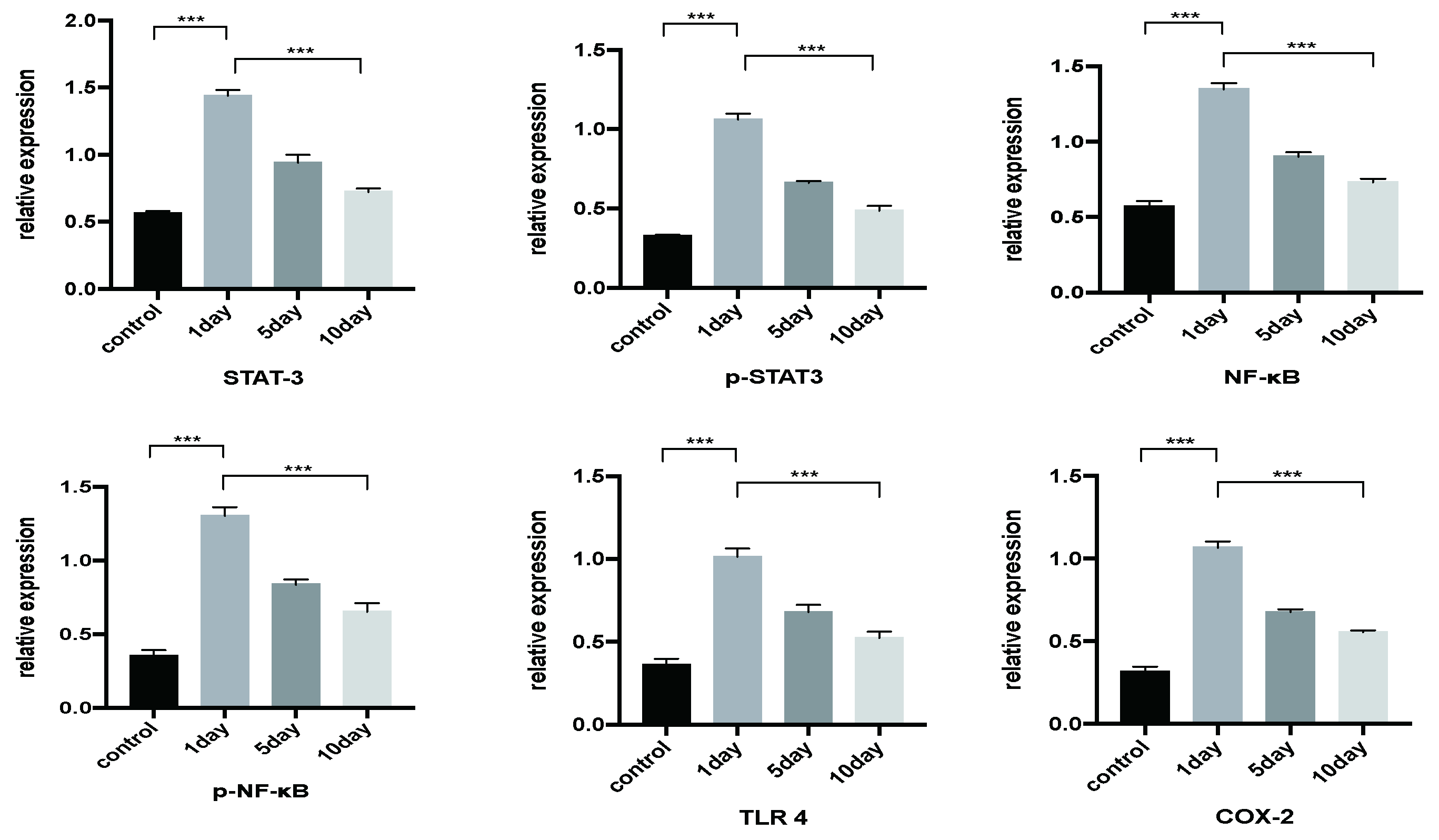

Supplement: Supplementary Figure 2 — Relative protein expression. Data are represented as the mean ± standard deviation (SD). ***p <0.001; * *p <0.01; *p <0.05. [file Image_2.tif]
